# Comparative genomic analysis of multiple mammary tumors from a single dog: whole-genome sequencing study

**DOI:** 10.1186/s13620-025-00311-5

**Published:** 2025-10-30

**Authors:** Keon Kim, Tae-Hoon Shin, Sin-Wook Park, Sang-Ik Park, Yoon Jung Do, Woong-Bin Ro, Chang-Min Lee

**Affiliations:** 1 Department of Veterinary Internal Medicine, College of Veterinary Medicine and BK21 FOUR program, Chonnam National University, Gwangju, 61186 Republic of Korea; 2 Department of Laboratory Animal Medicine, College of Veterinary Medicine, Veterinary Medical Research Institute, Jeju National University, Jeju, 63243 Republic of Korea; 3 Department of Veterinary Pathology, College of Veterinary Medicine, Chonnam National University, Gwangju, 61186 Republic of Korea; 4 Division of Animal Diseases & Health, Rural Development Administration, National Institute of Animal Science, Wanju-gun, 55365 Republic of Korea; 5 77 Yongbongro buk-gu, Gwangju, Republic of Korea

**Keywords:** Breast cancer, Canine, Mammary gland tumor, Next-generation sequencing, Whole-genome sequencing

## Abstract

**Background:**

Next-generation sequencing of canine spontaneous cancer is a powerful approach in both comparative oncology and veterinary genomics. We encountered a unique case with concurrent mammary tumors. Using whole-genome sequencing (WGS), we profiled the tumor-specific landscape of somatic mutations across multiple tumor subtypes, providing unprecedented evidence within an identical genetic background.

**Results:**

Of the seven mammary gland tumors (MGTs) isolated, two were diagnosed as benign and five as malignant. High-quality WGS (34.5X average sequencing depth, ≥ 20X coverage across 94.9% of the genome) on tumors and a blood sample revealed missense mutations in human breast cancer-related genes such as *BRCA2* and *TP53*. Furthermore, we found evidence of canine-specific somatic mutations depending on the tumor subtypes, including *HECTD4* in malignant tumors and *NIPBL* in epithelial-derived malignant tumors.

**Conclusions:**

This unique case study provides novel insights into the genomic heterogeneity, clonal evolution, and subtype-specific pathogenesis of naturally occurring canine MGTs. Despite some inherent limitations and potential for individual-specific variation, our results emphasize and guide future large-scale, longitudinal studies to further elucidate the clinical and biological significance of key somatic alterations.

**Supplementary Information:**

The online version contains supplementary material available at 10.1186/s13620-025-00311-5.

## Background

Mammary gland tumors (MGTs) are the most common tumors in unspayed and spayed female dogs [1]. Genetic predisposition as well as other factors, such as hormonal influences, diet, and obesity, are known to contribute to the risk of developing MGTs [2]. The breeds with an increased MGT risk can vary according to each study and geographic location. However, the vast majority of cases are diagnosed in small dogs such as Poodles, Pinschers, Dachshunds, Yorkshire Terriers, and Shih Tzus [3–5]. Although in general, cancer is considered a multifactorial disease, clustering of particular cancers in specific pedigrees suggests a genetic predisposition [6]. As the genetic diversity within a particular breed is generally lower than the overall diversity of the entire canine species, certain breeds may accumulate risk alleles over time due to limited gene flow [5].

With the sequencing of the entire canine genome and its close similarity to the human genome, the dog has become an appealing alternative model for cancer research [7]. For many gene families, especially those related to cancer, the similarities between dog and human gene sequences are much closer than those between mouse and human gene sequences [8]. Altered expression of oncogenes in these two species has also been observed, suggesting similar roles in carcinogenesis [9–11]. Indeed, various human studies have identified genes associated with hereditary cancer syndromes and an increased risk of breast cancer [12–19]. These genes are implied to potentially have similar functions in dogs with MGT. Furthermore, in addition to genetic factors, dogs and humans often share the same living environment, leading to common environmental carcinogens. Various studies have identified a correlation between environmental factors and the incidence of human breast cancer in patients who have dogs with MGTs [20, 21]. The genomic alterations identified in canine MGTs that parallel those observed in human breast cancer are summarized in Table 1.

To date, research on canine MGTs has revealed preclinical features that can be extended to human breast cancer and has focused mainly on comparative studies [25, 26]. Furthermore, existing studies on genetic mutations of MGTs in dogs have been mostly conducted based on genetic mutations found in human breast cancer rather than being specific to dogs. Therefore, identifying canine-specific genetic mutations associated with MGTs is necessary for comparative medicine with human breast cancer and to provide a clear understanding of the development of canine MGTs. Overcoming these challenges can be significantly aided by whole-genome analysis. The process of examining the entire genome enables the identification of all genetic mutations linked to a tumor and has the advantage of detecting specific mutations unique to the species. The first WGS analysis of MGTs in four large purebred dogs (a Golden Retriever, Siberian Husky, Standard Schnauzer, and Dalmatian) was recently conducted [27], and a genome-wide association study (GWAS) analysis has also been reported for Maltese, which is a breed with a high prevalence of MGTs [28].

Recent breakthroughs in genomic technologies have significantly advanced veterinary oncology, particularly in the field of canine cancer genomics. Several studies have consistently demonstrated a remarkable resemblance between canine cancer genomics and their corresponding human counterparts, thereby affirming the dog as an invaluable spontaneous model for human oncology [29]. Notably, a large-scale oncogenomic study on canine mammary tumors, employing whole-exome and RNA sequencing, revealed striking similarities with human breast cancer, as evidenced by a similar PIK3CA mutation frequency, shared aberrations in the PI3K-Akt pathway, and common driver mutations including TP53 and PTEN [30]. Another whole-genome sequencing study on 12 dog patients with spontaneous MGTs found a more similar genomic profile in simple carcinoma, compared to complex carcinoma, to human breast cancer [31]. Table 1 summarizes the current knowledge on genomic alterations shared between human and canine mammary tumors. Concurrently, these studies also provided canine-specific molecular evidence, including a relative lack of ERBB2 amplification in canine MGTs and the dominance of epigenetic aberrations over genomic alterations in complex carcinoma, respectively. Collectively, canine models with spontaneous MGTs, unlike induced or xenografted rodent models, serve as a robust preclinical model for understanding human tumor biology, as well as a critical platform for comparative oncology aimed at identifying species-specific differences for veterinary care.

**Table 1:** A summary of currently documented genomic alterations common to canine MGT and human breast cancer

Category of Alteration	Gene	Specific Alteration and homology	References
Somatic Point Mutation	*PIK3CA*	Homologous gain-of-function hotspot mutations in the helical (e.g., E545K) and kinase (e.g., H1047R) domains	[22]
	*TP53*	Inactivating mutations (missense, nonsense, frameshift) in the DNA-binding domain	[22]
	*BRCA1/BRCA2*	Germline variants conferring increased risk; somatic mutations in sporadic tumors	[23]
	*KRAS*	Activating mutations	[23]
	*NF1/SF3B1*	Inactivating mutations	[23]
	*EGFR/ATM/CHEK2*	Various mutations	[23]
Copy Number Gain (Amplification)	*MYC*	Recurrent amplification of the gene locus	[24]
	*ERBB2 (HER2)*	Gene amplification leading to protein overexpression	[22]
Copy Number Loss (Deletion)	*PTEN*	Deletion of the gene locus, including homozygous deletions	[24]
	*CDKN2A/B*	Co-deletion or homozygous loss of the gene locus	[9]

Next-generation sequencing (NGS) is a completely new sequencing technology known as a powerful tool for detecting variants within an individual’s genome [32, 33]. Recently, researchers have attempted to apply NGS-based WGS analysis to various research fields such as oncology in human medicine [34, 35]. However, in dogs, there are very few NGS-based WGS analyses of any disease. In this study, we aim to explore somatic mutations in canine MGTs through a unique case with seven distinct tumors through WGS studies using the NGS technique. To date, previous WGS studies of canine MGTs have primarily focused on identifying inherited germline variants across multiple breeds [27, 36]. As a result, investigating somatic genomic alterations that drive the development of multiple heterogeneous tumors within a single individual has been highly limited in spontaneous tumor models. Through this study, we seek to identify mutations associated with malignancy and specific histopathological subtypes, thereby providing genetic insights that may distinguish early initiating events from later events driving tumor progression.

## Methods

**Fig. 1:**
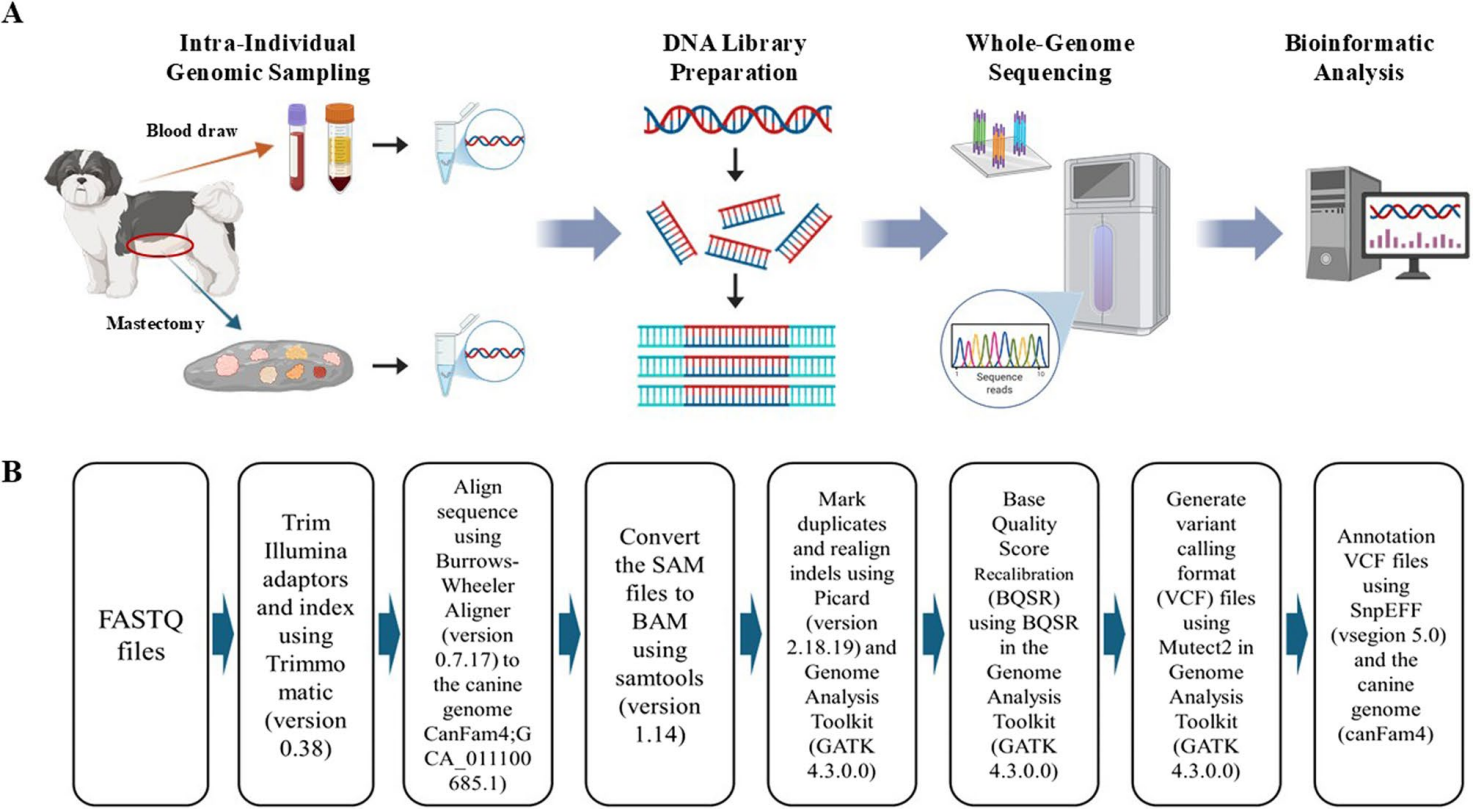
Schematic outline for whole-genome sequencing and bioinformatic analysis employed in the study. **A** Graphical representation of intra-individual whole-genome sequencing workflow on mammary tumors and blood using the high-quality NovaSeq X platform. All the collected genomic DNAs and constructed libraries underwent rigorous quality control and verification to proceed with the next step. The illustration was created with BioRender.com. **B** Pipelines used for bioinformatic analysis of the WGS data

1. The aim of the study
  In this study, we performed WGS analysis on seven different types of MGTs and a blood sample from a single dog. As this is a single case, genome identity could be ensured, and it was hypothesized that genetic variations across each neoplasm represent somatic mutations specific to each histopathological type. Through the WGS analysis, we sought to identify somatic mutations across the entire genome for each type of tumor. For any non-synonymous variations observed, in silico validation was conducted to predict the risk associated with mutations at the protein level. In addition, we compared alignments to human protein sequences to identify known genetic variations in existing human databases. This is the first study to perform WGS analysis in seven distinct tumors and whole blood derived from a single dog.
2. Sample collection and Preparation
  Mammary gland neoplastic tissues were obtained by bilateral mastectomy. A venous blood sample was collected and added to an ethylenediaminetetraacetic acid (EDTA) anticoagulant tube, and the tube was stored at −80 °C. Tissue samples were stored at −80 °C until genomic DNA extraction. Seven neoplastic tissues were thawed and homogenized just before DNA extraction. Genomic DNA was extracted from homogenized tumor tissue and buffy coat using the QIAmp DNA mini kits and QIAmp DNA blood mini kits (Qiagen, Germany) according to the manufacturer’s instructions. DNA concentrations were measured using a Nabi UV/Vis nano spectrophotometer (MicroDigital Co., Ltd., South Korea). The A260/A280 ratio of all extracted samples was within the normal range of 1.7–1.9.
3. Preoperative examination
  As a preoperative evaluation for mastectomy, blood examination and diagnostic imaging studies were performed to assess the health status for generalized anesthesia. The blood tests included a complete blood count (CBC), serum chemistry covering 17 parameters including albumin, albumin/globulin ratio, alkaline phosphatase(ALP), alanine transaminase(ALT), amylase, blood urea nitrogen (BUN), creatinine (CREA), BUN/CREA ratio, calcium, cholesterol, gamma-glutamyltransferase (GGT), globulin, glucose, lipase, phosphate, total bilirubin, total protein), blood gas analysis, and electrolytes profiling. Hematologic parameters were measured using a CBC analyzer (Procyte Dx analyzer, IDEXX Veterinary Diagnostics Co., USA). Serum chemistry panels were analyzed using the serum chemistry analyzer (Catalyst Dx Chemistry Analyzer, IDEXX Veterinary Diagnostics Co., USA). The results of blood gas and electrolytes were quantified using a blood gas and electrolytes analyzer (pHOx Ultra, Nova Biomedical Co., South Korea). The diagnostic imaging comprised thoracic radiography, abdominal radiography, and abdominal ultrasonographic evaluation. Diagnostic imaging evaluation was performed using the digital radiography system (TITAN 2000v, Gemss-Medical, South Korea) and ultrasound equipment (Prosound α7, Hitachi-Aloka, Wallingford, USA).
4. Histopathology
  After mastectomy, neoplastic tissues were fixed in 10% neutralized formalin solution. The pathological material was processed with SHANDON Pathcentre™ (Thermofisher Scientific, USA) and embedded into paraffin. Section (3-µm thick) from the formalin-fixed paraffin-embedded blocks were stained with hematoxylin and eosin. All tissue samples were evaluated and diagnosed by board-certified veterinary pathologists. The histological grade was assessed according to the grading system for epithelial-derived tumors, which is known as the Peña system [37].
5. Sequencing and quality control
  Genome sequencing was performed on Illumina NovaSeq X. Paired FASTQ files were obtained for each sample; the quality of the raw FASTQ files was determined using FASTQC. Samples were classified as pass if the Q30 read exceeded 65% and the survived reads were above 70%.
6. Processing of WGS data
  After assuring quality files, these sequencing data were carried through an in-house bioinformatics canine pipeline that was adapted from the Genome Analysis Toolkit (GATK) best practices bioinformatics pipeline (Fig. 1). In brief, each sample file had Illumina adapters trimmed using the program Trimmomatic [38]. Sequences were aligned to the canFam4(UU_Cfam_GSD_1.0) reference genome with BWA mem [39, 40]. Duplicate reads were marked and eliminated using a Picard tool form version 2.25.6-0.6.6; then, indels were realigned, and base quality scores were recalibrated using Base Quality Score Recalibrator (BQSR). In addition, using GATK, coverage was calculated using the Depth of Coverage tool, and genomic variant calling format files were generated using Mutect2. SNPeff [41] was used to annotate the VCF files using gene prediction from canFam4.

(7) In silico verification and isolation of coding variants within orthologs of human genes for breast cancer
  Major genes associated with breast cancer were selected for comparison coding variants within orthologs of human genes. Based on human medical research, most genes reported to be associated with hereditary cancer syndrome and an increased risk of breast cancer were chosen [12–19]. For coding variants in which a missense mutation was identified, the risk of the amino acid substitution affecting the protein structure and function was evaluated. The risk prediction was performed using in silico validation with PolyPhen-2 (http://genetics.bwh.harvard.edu/pph2/). Furthermore, coding variants within the selected genes were identified through WGS, and canine protein sequences were compared to the human sequences through BOSS water alignment (https://www.ebi.ac.uk/Tools/psa/emboss_water/) following the guidance in a previous report [27]. Corresponding human amino acids of each coding variant were evaluated to see if a human mutation was identified in that position using the ClinVar database [42].

## Results

**Fig. 2:**
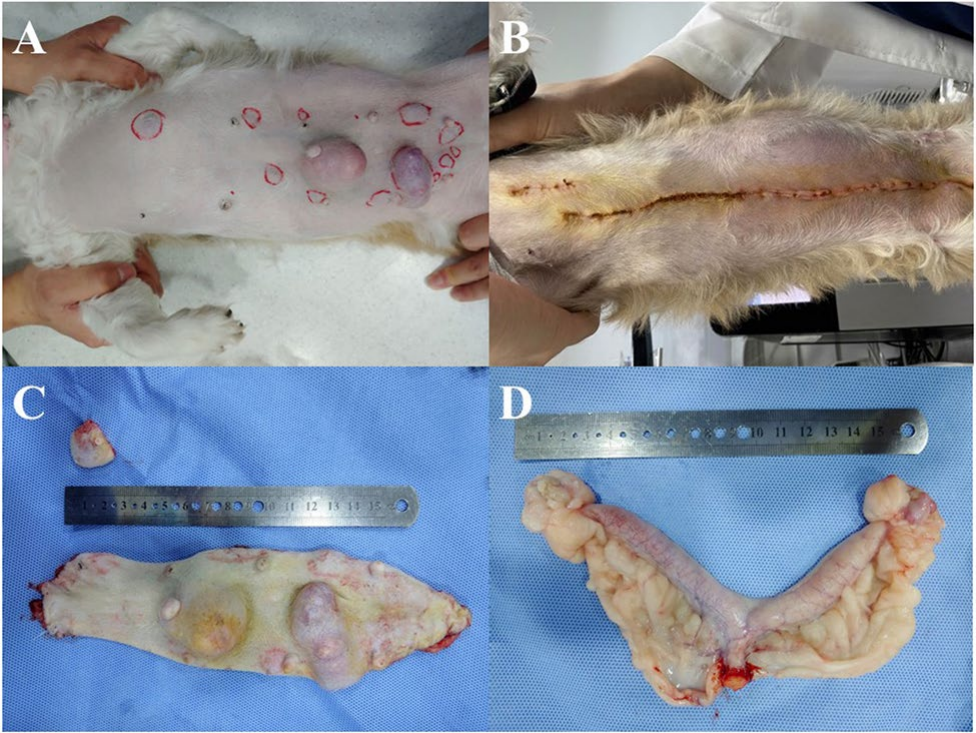
Gross appearance of MGTs in a single dog: pre- and post-surgical observations. **A** Pre-surgical distribution of multiple MGTs. **B** Appearance after partial mastectomy. **C** Excised neoplastic mammary gland tissues. A total of seven MGTs were identified, and one mass-like lesion was an umbilical hernia. **D** Excised ovaries and uterus from the concurrent ovariohysterectomy (OHE)
  A regional mastectomy including the abdominal and inguinal mammary glands was performed, and a separate simple mastectomy was conducted for the left thoracic MGT. An ovariohysterectomy (OHE) was performed using conventional methods. The extracted neoplastic tissues were submitted to veterinary histopathologists for evaluation. The histopathological results for the seven MGTs are described in Fig. 3; Table 2 and Supplementary Table 1.

1. Case presentation
  A 12-year-old, intact female Shih Tzu weight 5.1 kg was presented to the animal hospital with multiple MGTs located within entire abdomen (Fig. 2). On physical examination, an umbilical hernia was also found. A CBC showed a prominent leukocytosis (white blood cell (WBC) count = 28.6 × 10^9^/L, reference interval (RI): 6–17 × 10^9^/L) and thrombocytosis (platelet (PLT) count = 838 × 10^9^/L, RI: 200–500 × 10^9^/L). The serum biochemistry results did not reveal any significant variations except for mild azotemia (blood urea nitrogen (BUN) = 35 mg/dL, RI: 9.2–29.2 mg/dL) and hypercholesteremia (cholesterol (CHOL) = 313 mg/dL, RI: 111–312 mg/dL). Blood gas analysis suggested that this patient had respiratory acidosis. Diagnostic imaging including radiography and abdominal ultrasound showed no evidence of metastasis.

**Fig. 3:**
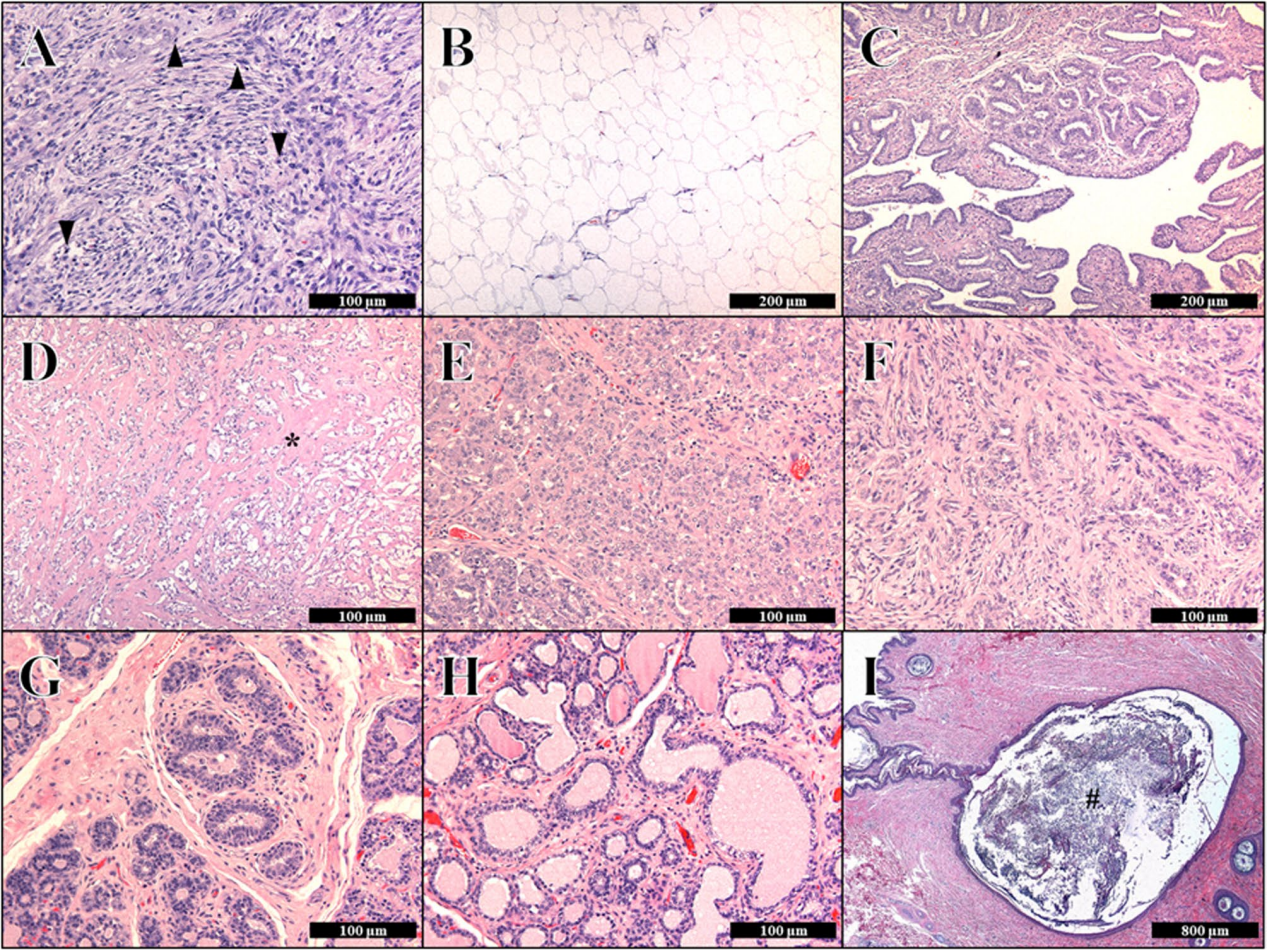
Histopathological evaluation of seven mammary gland tumors in a dog Seven mammary gland tumor lesions in hematoxylin and eosin stain. **A** First mammary gland: malignant myoepithelioma showing frequent mitotic figures (arrows); Bar = 100 μm. **B** Second mammary gland: lipoma with well-differentiated lipocytes; Bar = 200 μm. **C** Third mammary gland: tubulopapillary carcinoma; Bar = 200 μm. **D**,** E** Fourth mammary gland: tubular carcinoma with bone formation with osteoid formation (asterisk); Bar = 100 μm. **F** Fifth mammary gland: complex carcinoma composed of two populations of neoplastic cells, including tubular epithelial and myoepithelial cells. **G** Sixth mammary gland: in situ carcinoma; Bar = 100 μm. **H, I** Seventh mammary gland: simple adenoma and follicular cyst showing severe accumulation of keratin (hash tag); Bar = 100 μm and 800 μm, respectively

**Table 2:** Histopathological diagnosis and post-trimming sample quality parameters

Sample	Histopathologic diagnosis	Q_30_ (%)	Survived Reads (%)
T1	Malignant myoepithelioma (grade III malignancy)	94.12	88.52
T2	Lipoma	93.19	86.11
T3	Tubulopapillary carcinoma (grade I malignancy)	93.4	86.61
T4	Tubular carcinoma (grade I malignancy)	94.3	88.69
T5	Complex carcinoma (grade II malignancy)	94.35	88.35
T6	In situ carcinoma (grade I malignancy)	94.52	88.52
T7	Simple adenoma	94.15	87.89
Blood	(-)	94.33	88.26
2. Sequencing and annotation
  All eight samples exhibited mean Q30 reads above 94% and survival reads above 87.8% (Table 2). The WGS results of the eight samples achieved an average sequencing depth of 34.5X (Table 3). On average, 99.2% of the reads were correctly aligned with the reference genome, which led to genome coverage of 99.5%, 99.2%, 98.6%, and 94.9% at minimum depths of 1X, 5X, 10X, and 20X, respectively. After variant calling, the total chromosome length was confirmed as 2,353,542,698, with a total of 2,432,180 variants identified. Out of a total of 2,432,180 variants, single-nucleotide variations (SNVs) accounted for the majority with 1,769,568 variants, followed by 420,344 deletions, 219,455 insertions, and 22,813 multi-nucleotide variations (MNVs).

**Table 3:** Results for the depth of coverage from WGS analysis

#Coverage	Number of Mapped Reads to canFam4	% of Reads Mapped to canFam4	Average Sequencing Depth	% of bases covered greater than or equal to								
				1X	5X	10X	15X	20X	25X	50X	75X	100X
**T1**	686,693,553	99.93%	35.6	99.5	99.1	98.5	97.4	94.3	87.4	7.4	0.3	0.1
**T2**	591,380,370	99.86%	31	99.5	99.2	98.6	97.4	94.1	83.4	0.8	0.1	0.1
**T3**	808,356,815	99.94%	41	99.6	99.2	98.9	98.4	97.5	95.7	15.9	0.4	0.1
**T4**	695,242,412	99.93%	36.9	99.6	99.2	98.8	98.1	96.7	93.3	5.3	0.3	0.1
**T5**	731,897,360	99.93%	38.7	99.5	99.2	98.8	98.1	97.0	94.5	8.9	0.3	0.1
**T6**	526,441,566	99.93%	28.2	99.5	99.1	98.3	96.5	90.3	72.2	0.5	0.1	0.1
**T7**	607,410,788	99.91%	32.2	99.5	99.1	98.5	97.4	94.7	86.4	1.2	0.2	0.1
**Blood**	604,479,112	99.94%	32	99.6	99.2	98.6	97.5	94.7	86.0	1.1	0.	0.1
**Mean**	656,487,747	99.92%	34.5	99.5	99.2	98.6	97.6	94.9	87.3	5.1	0.2	0.1
3. Analysis of SNVs associated with somatic mutations
  A Genetic alterations in tumors
    Initially, using the genomic sequence of the blood sample as a reference, the WGS analysis revealed 817,589 variations in the other seven MGT neoplastic tissues. According to the annotation results, over 80% of the variations were found to be distributed in intron and intergenic regions. A considerable number of critical variations within the transcript region, presumed to have implications for protein synthesis, were observed. The outcomes of these observations are comprehensively delineated in Supplementary Table 2.
  B Genetic alterations in tumors with malignancy
    Variations were identified exclusively in five malignant tumors (T1, T3, T4, T5, T6) that were distinct from normal blood and benign tumors. A total of 1,191 variations were detected, and after excluding those located in intergenic regions, 739 SNVs were confirmed. The majority of these variations were distributed within introns (509/739); 140 were located upstream of genes, and 63 were located downstream of genes. Table 4 displays the positions of significant variations, excluding those that are synonymous variants and related to the transcription of genes. In particular, a missense mutation in the *HECTD4* gene (c.5863G > A) showed that it was “probably damaging” in the in silico verification.

**Table 4:** Information related to the major SNVs identified only in malignant tumors and epithelial-derived malignant tumors (carcinoma)

Malignant MGTs								
#Chrom	Pos	Ref	Alt	Gene	N_change	AA_change	Genomic feature	Polyphen score
7	12,956,632	TA	T	*KCNK2*	c.*96delA	.	3_prime_UTR	NA
7	37,546,899	T	C	*AHCTF1*	c.1360 A > G	p.Ile454Val	Missense	0.001 (Benign)
13	59,673,140	C	A	*YTHDC1*	c.−188 C > A	.	5_prime_UTR	NA
14	38,296,146	T	G	*GSDME*	c.*341A > C	.	3_prime_UTR	NA
14	42,890,496	G	A	*FKBP14*	c.*340C > T	.	3_prime_UTR	NA
18	6,758,450	C	T	*HECW1*	c.*2232G > A	.	3_prime_UTR	NA
26	10,154,058	C	T	*HECTD4*	c.5863G > A	p.Ala1955Thr	Missense	0.996 (Probably damaging)

**Epithelial-derived malignant MGTs**								
**#Chrom**	**Pos**	**Ref**	**Alt**	**Gene**	**N_change**	**AA_change**	**Genomic feature**	**Polyphen score**
2	74,994,954	G	GC	*SRSF10*	c.*1581dupC	.	3_prime_UTR	NA
3	38,429,578	T	G	*MPHOSPH10*	c.*1475A > C	.	3_prime_UTR	NA
4	72,588,058	C	A	*NIPBL*	c.1405G > T	p.Val469Leu	Missense	0.628 (Possibly damaging)
9	25,824,909	G	A	*KAT7*	c.*169G > A	.	3_prime_UTR	NA
12	9,971,351	G	A	*UNC5CL*	c.*857C > T	.	3_prime_UTR	NA
17	60,458,375	CT	C	*ENSA*	c.*722delA	.	3_prime_UTR	NA
18	45,983,259	G	A	*MUC5B*	c.9925G > A	p.Gly3309Arg	Missense	Unknown
22	36,445,247	G	A	*SLITRK1*	c.1381 C > T	p.Leu461Phe	Missense	0.070 (Benign)
24	24,534,774	G	A	*MYH7B*	c.−273G > A	.	5_prime_UTR	NA
X	42,578,096	G	GT	*PPP1R3F*	c.−1987dupT	.	5_prime_UTR	NA
  C Genetic alterations in epithelial-derived neoplasia
    We identified variations that appeared exclusively in four tissues classified as epithelial-derived malignant tumors (T3, T4, T5, T6) that were different from other samples. A total of 1,112 variations were identified, excluding 412 intergenic variations and 491 variations in introns. Among the remaining 209 variations, non-synonymous variants and those located in regions critical to gene transcription are presented in Table 4. In the in silico verification, a missense mutation in the *NIPBL* gene (c.1405G > T) showed that it was “possibly damaging”.
4. Variant analyses associated with breast cancer for human ortholog genes
  The following seventeen genes with human orthologs associated with breast cancer in dogs were selected: *BRCA1*,* BRCA2*,* TP53*,* STK11*,* CDH1*,* PTEN*,* AKT*,* HER2*,* IGF1*,* ERBB2*,* SHBG*,* AR*,* ESR1*,* PGR*,* CDKN2A*,* RB1*, and *PALB2*. According to WGS analysis, a total of 789 SNVs were identified at positions related to these genes. Of these variants, 28.14% (222/789) were distributed in intergenic locations, and 57.8% (456/789) were located in introns. Excluding variants found in splice regions and upstream and downstream positions, 33 were identified. Among these, 9 were confirmed to be synonymous variants, and the remaining 24 non-synonymous variants are presented in Table 5. The genotypes for each MGT tissue and the blood sample, with respect to the 24 variants, are also presented in Table 6.

**Table 5:** Information related to the major SNVs identified in human ortholog genes associated with breast cancer

#Chrom	Pos	Ref	Alt	Gene	N_change	AA_change	Genomic feature	Polyphen score
1	42,773,173	G	GT	*ESR1*	c.*1807dupT	.	3_prime_UTR	NA
1	42,771,789	C	A	*ESR1*	c.*419C > A	.	3_prime_UTR	NA
5	81,347,396	C	A	*CDH1*	c.2281G > T	p.Glu761*	stop_gained	NA
5	81,368,793	ATGG	A	*CDH1*	c.387_389delCCA	p.His130del	disruptive_ inframe_deletion	NA
5	81,342,226	T	C	*CDH1*	c.*1240A > G	.	3_prime_UTR	NA
5	81,342,279	G	GA	*CDH1*	c.*1186dupT	.	3_prime_UTR	NA
5	81,342,596	T	A	*CDH1*	c.*870A > T	.	3_prime_UTR	NA
5	81,342,766	G	T	*CDH1*	c.*700C > A	.	3_prime_UTR	NA
5	81,343,345	T	C	*CDH1*	c.*121A > G	.	3_prime_UTR	NA
5	81,343,067	TAC	T	*CDH1*	c.*397_*398delGT	.	3_prime_UTR	NA
5	32,746,330	C	T	*SHBG*	c.457 C > T	p.Arg153Cys	missense	0.625 (Possibly damaging)
5	32,771,285	A	G	*TP53*	c.685T > C	p.Ser229Pro	missense	1.000 (Probably damaging)
6	22,461,625	T	G	*PALB2*	c.1439T > G	p.Leu480Arg	missense	0.938 (Possibly damaging)
9	22,713,702	C	T	*ERBB2*	c.3565G > A	p.Val1189Ile	missense	0.018 (Benign)
11	41,339,546	C	A	*CDKN2A*	c.376G > T	p.Ala126Ser	missense	0.021 (Benign)
11	41,342,858	CTCCT	C	*CDKN2A*	c.−46_−43delAGGA	.	5_prime_UTR	NA
11	41,364,102	G	T	*CDKN2A*	c.−78 C > A	.	5_prime_UTR	NA
20	58,046,465	G	T	*STK11*	c.824 C > A	p.Pro275Gln	missense	1.000 (Probably damaging)
20	58,039,906	G	A	*STK11*	c.*340C > T	.	3_prime_UTR	NA
21	571,913	C	A	*PGR*	c.1888 C > A	p.Leu630Ile	missense	1.000 (Probably damaging)
25	7,822,932	C	A	*BRCA2*	c.6686G > T	p.Cys2229Phe	missense	0.969 (Probably damaging)
25	7,825,345	T	G	*BRCA2*	c.4273 A > C	p.Thr1425Pro	missense	0.792 (Possibly damaging)
25	7,839,968	A	G	*BRCA2*	c.308T > C	p.Ile103Thr	missense	0.361 (Benign)
X	52,313,056	C	A	*AR*	c.1813 C > A	p.Leu605Ile	missense	0.990 (Probably damaging)

**Table 6:** Sequencing of variants in all MGT tissues and blood identified in human ortholog gene associated with breast cancer

#Chrom	Pos	Ref	Alt	Gene	T1	T2	T3	T4	T5	T6	T7	Blood
1	42,773,173	G	GT	*ESR1*	G/G	G/GT	G/GT	G/G	G/G	G/G	G/G	G/GT
1	42,771,789	C	A	*ESR1*	C/A	C/C	C/C	C/C	C/A	C/C	C/C	C/C
5	81,347,396	C	A	*CDH1*	C/C	C/A	C/A	C/A	C/C	C/C	C/C	C/C
5	81,368,793	ATGG	A	*CDH1*	ATGG/A	ATGG/A	ATGG/A	ATGG/A	ATGG/A	ATGG/A	ATGG/A	ATGG/ ATGG
5	81,342,226	T	C	*CDH1*	T/C	T/C	T/C	T/C	T/C	T/C	T/C	T/T
5	81,342,279	G	GA	*CDH1*	G/GA	G/GA	G/GA	G/GA	G/GA	G/GA	G/GA	G/G
5	81,342,596	T	A	*CDH1*	T/A	T/A	T/A	T/A	T/A	T/A	T/A	T/T
5	81,342,766	G	T	*CDH1*	G/T	G/T	G/T	G/T	G/T	G/T	G/T	G/G
5	81,343,345	T	C	*CDH1*	T/C	T/C	T/C	T/C	T/C	T/C	T/C	T/T
5	81,343,067	TAC	T	*CDH1*	TAC/TAC	TAC/TAC	TAC/TAC	TAC/TAC	TAC/T	TAC/TAC	TAC/T	TAC/TAC
5	32,746,330	C	T	*SHBG*	C/T	C/T	C/T	C/T	C/T	C/T	C/T	C/C
5	32,771,285	A	G	*TP53*	A/G	A/A	A/A	A/A	A/A	A/A	A/A	A/A
6	22,461,625	T	G	*PALB2*	T/G	T/G	T/G	T/G	T/G	T/G	T/G	T/T
9	22,713,702	C	T	*ERBB2*	C/T	C/T	C/T	C/T	C/T	C/T	C/T	C/C
11	41,339,546	C	A	*CDKN2A*	A/A	A/A	A/A	A/A	A/A	A/A	A/A	C/C
11	41,342,858	CTCCT	C	*CDKN2A*	C/C	C/C	C/C	C/C	C/C	C/C	C/C	CTCCT/ CTCCT
11	41,364,102	G	T	*CDKN2A*	G/G	G/G	G/G	G/T	G/G	G/G	G/G	G/G
20	58,046,465	G	T	*STK11*	G/G	G/G	G/T	G/G	G/G	G/G	G/G	G/G
20	58,039,906	G	A	*STK11*	G/A	G/A	G/A	G/A	G/A	G/A	G/A	G/G
21	571,913	C	A	*PGR*	C/C	C/A	C/C	C/C	C/C	C/C	C/C	C/C
25	7,822,932	C	A	*BRCA2*	C/A	C/A	C/A	C/A	C/A	C/A	C/A	C/C
25	7,825,345	T	G	*BRCA2*	T/G	T/G	T/G	T/G	T/G	T/G	T/G	T/T
25	7,839,968	A	G	*BRCA2*	A/G	A/G	A/G	A/G	A/G	A/G	A/G	A/A
X	52,313,056	C	A	*AR*	C/C	C/A	C/A	C/C	C/C	C/C	C/C	C/C

Among these 24 variants, 15 were exclusively identified with somatic mutations in all MGT tissues. In the *CDH1* gene, five 3’-UTR variants and one disruptive in-frame deletion were identified in all the MGTs. In the *BRCA2* gene, three missense mutations were identified in all the MGTs. Among these mutations, one missense mutation followed by a coding variant (c.6686G > T) showed a high risk of amino acid substitution (p.Cys2229Phe).

Among these 24 variants, 9 identified with somatic mutations in several MGT tissues. One stop-gained variant in the *CDH1* gene was also identified in several tumors (T2, T3, T4). A missense mutation of the *TP53* gene was also identified exclusively in a malignant myoepithelioma (grade 3), and the amino acid change (p.Ser229Pro) was confirmed as “probably damaging”.

**Fig. 4:**
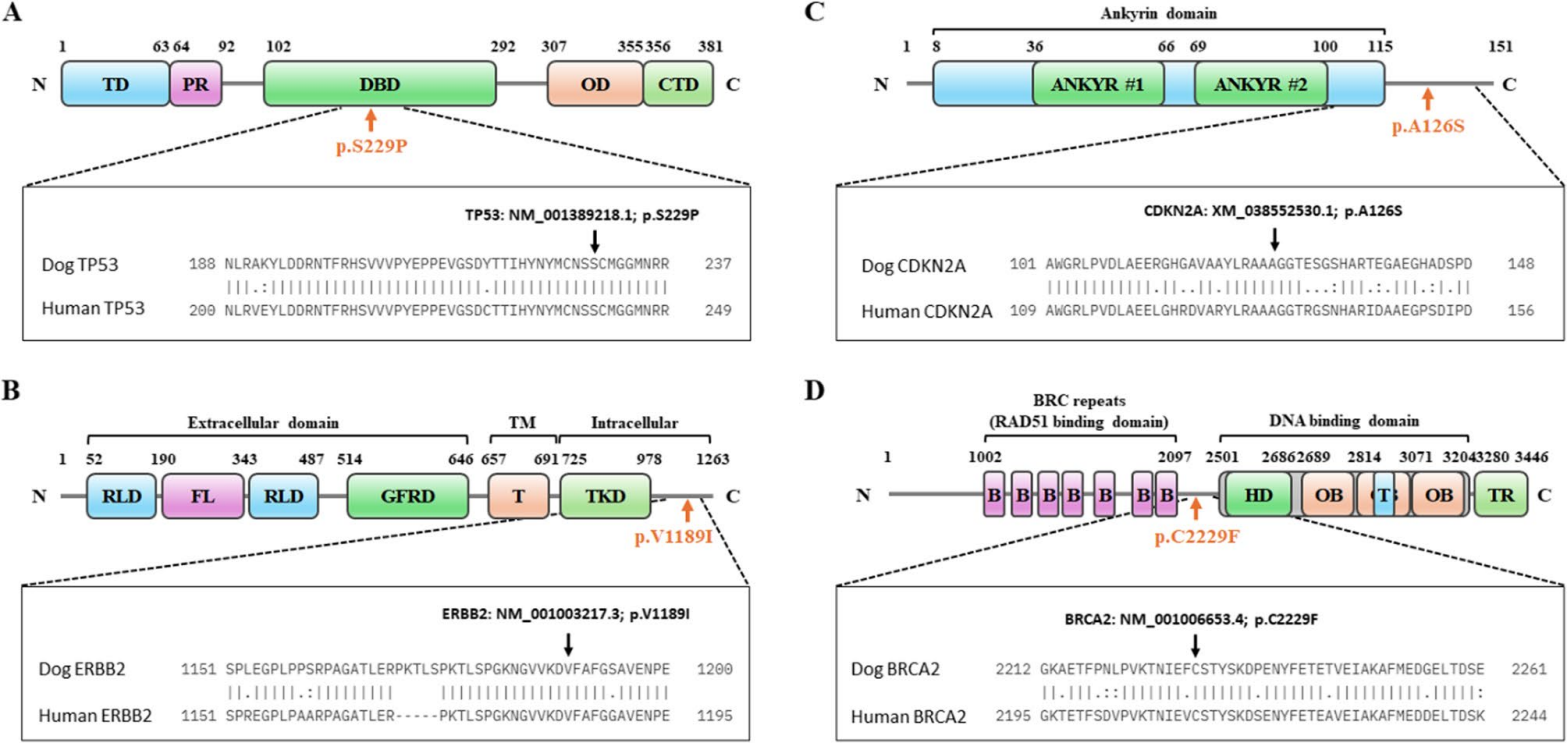
Dog and human protein alignments for identical non-synonymous variants previously reported in the ClinVar database. The locations of four major non-synonymous mutations, including TP53, ERBB2, CDKN2A, and BRCA2, are depicted in the corresponding domain structure. The boxes below each panel indicate the translated amino acid sequences of the regions around the mutation site, the NCBI RefSeq ID used as the reference transcript, and the human sequence equivalents. **A** TP53. N = N-terminus; TD = transactivation domain; PR = Proline-rich domain; DBD = DNA-binding domain; OD = oligomerization domain; CTD = C-terminal regulatory domain; C = C-terminus. **B** ERBB2. RLD = Receptor L-domain; FL = Furin-like cysteine-rich domain; GFRD = Growth factor receptor domain IV; TM = Transmembrane domain; T = Epidermal growth factor receptor-like, transmembrane-juxtamembrane segment; TKD = Serine-threonine/tyrosine-protein kinase, catalytic domain; CT = C-terminal tail. **C** CDKN2A. ANKYR = Ankyrin repeat. **D** BRCA2. B = BRCA2 repeats; HD = helical domain; OB = oligonucleotide/oligosaccharide-binding domain; T = tower domain; TR = TR2 region, RAD51 binding domain

As shown in Fig. 4, amino acid variations were aligned with the corresponding human protein sequences for each gene. Based on the aligned human amino acid sequences, we verified whether the amino acid variations identified were also previously reported in humans using the ClinVar database. According to the database, identical amino acid mutations in the *CDH1*, *TP53*,* ERBB2*,* CDKN2A*, and *BRCA2* genes have been reported in humans. Notably, a stop-gained variant in the *CDH1* gene (Canine: p.Glu761*) is known to act as “pathogenic” in human breast cancer (Human: p.Glu758*). Corresponding amino acid substitutions in the *TP53* and *BRCA2* genes showed that they were “likely pathogenic” and had “conflicting classifications of pathogenicity”, respectively. The aligned sequences of amino acids for the four genes and the mutations reported in ClinVar are presented in Fig. 4; Table 7, respectively. In addition, other similar mutations reported at the locations of amino acid changes are listed in Supplementary Table 3.

**Table 7:** Identical variants in dogs corresponding to human amino acids matching the major variants reported in the clinvar database

#Chrom	Pos	Gene	AA_change (Canine)	AA_change (Corresponding human)	Mutation ID	Classification	Condition	Reference
5	81,347,396	*CDH1*	p.Glu761*	p.Glu758*	532,441	Pathogenic	CDH1-related diffuse gastric and lobular breast cancer syndrome	ClinGen CDH1 ACMG Specifications V3.1
5	32,771,285	*TP53*	p.Ser229Pro	p.Ser241Pro	376,664	Likely pathogenic	Breast neoplasm	PMID: 26,619,011 [43]
9	22,713,702	*ERBB2*	p.Val1189Ile	p.Val1184Ile	1,439,345	Uncertain significance	Not provided	PMID: 28,492,532 [44]
11	41,339,546	*CDKN2A*	p.Ala126Ser	p.Ala134Ser	532,288	Uncertain significance	Familial melanoma	PMID: 28,492,532 [44]
25	7,822,932	*BRCA2*	p.Cys2229Phe	p.Cys2212Phe	630,060	Conflicting classifications of pathogenicity	Hereditary cancer-predisposing syndrome	PMID: 25,741,868 [45] PMID: 31,911,673 [46]

## Discussion

To date, most genomic studies on canine MGTs have primarily focused on germline mutations to examine breed-specific predisposition or on somatic mutations to investigate molecular similarities to human breast cancer, commonly utilizing single tumor samples from different individuals of various breeds. Indeed, Husky et al. revealed that germline mutations in *BRCA2* and *STK11* were associated with the risk of MGTs not only in humans but also within specific canine breeds through a WGS on 14 purebred dogs with mammary tumors from four breed-specific pedigrees [27]. While these findings provided important evidence on the genetic predisposition of canine MGTs, a comprehensive analysis of somatic mutations associated with tumor progression, recurrence, and prognosis has not yet been fully investigated. We present here the first direct comparative analysis of somatic mutations across multiple subtypes of canine MGTs within the same individual. This novel approach enables a more precise assessment of tumor-specific mutational landscapes under a shared genetic background. Through a unique case of a single dog bearing seven distinct mammary tumors, we hypothesized that by identifying somatic mutations associated with malignancy and specific histopathological subtypes, we could gain genetic insights into early versus late genomic events involved in tumor progression. Each tumor sample obtained via mastectomy at the time of dagnosis was sequenced under conditions of better parameters (an average sequencing depth of 34.5X and a mean coverage of 94.9% at ≥ 20X) than reported in previous studies, yielding highly reliable SNV calls. Given the importance of sequencing quality in accurate profiling of the mutational landscape of intra-tumoral heterogeneity, the improved resolution attained here may support the high validity of the somatic variants identified, compared to previous WGS studies in this field. Our findings affirmed the relevance of known human breast cancer-associated genes in canine MGTs, further supporting the translational value of dogs as preclinical models. Importantly, we also identified potential canine-specific oncogenic drivers, such as *HECTD4* in malignant and *NIPBL* in epithelial-derived subtypes, that have been scarcely reported in human breast cancers, underscoring the significance in veterinary contexts.

In this case, seven MGTs were identified during a physical examination, leading to mastectomy, and histopathological examination revealed different results for each tumor. WGS was successfully performed on all seven MGT tissues and blood derived from this patient. A high level of sequencing depth and base coverage was achieved. As this was a single case, it was assumed that the genetic information for each sample would be completely consistent, and it was possible to identify specific somatic variations in each tumor. Most of the mutations that were commonly found exclusively in the tumor tissues were identified in intron and intergenic regions, while some occurred at multiple overlapping locations. Among the variations commonly found solely in malignant MGTs, seven genes associated with non-synonymous mutations at critical transcriptional sites were identified, with the *HECTD4* gene mutation specifically predicted to be “probably damaging” at the protein level. Among the SNVs that were exclusively identified in epithelial-derived malignant MGTs, ten genes associated with non-synonymous mutations at critical transcriptional sites were confirmed. Notably, a missense mutation in the *NIPBL* gene was predicted to be “possibly damaging.” Among the seventeen genes with human orthologs associated with breast cancer, mutations in the *CDH1*,* SHBG*,* PALB2*,* ERBB2*,* CDKN2A*,* STK11*, and *BRCA2* genes were identified in all the MGT tissues but not in the blood sample. A point mutation in the *TP53* gene was identified solely in malignant myoepithelioma (grade 3) among all the MGT tissues. Furthermore, corresponding amino acid substitutions in the *TP53*,* ERBB2*,* CDKN2A*, and *BRCA2* genes were confirmed in the human database.

In this study, WGS was performed on eight samples, with the individual average sequencing depth ranging from 28.2 to 41X and an overall average of 34.5X. This is significantly higher than the average sequencing depth of 26.0X reported in only one WGS study of MGT [27]. Aiming for a minimum average sequencing depth of 20X, all samples met this criterion, with all but one exceeding 30X. On average, 94.9% of the genome was covered by at least 20X. Similarly, this base coverage was much higher than the 75.6% achieved at 20X in a previous study [27]. The high sequencing depth achieved in this study (mean coverage of 34.5X) and the genome coverage (94.9% at ≥ 20X) were critical for the reliable detection of somatic SNVs. Such data quality is essential for accurately profiling the mutational landscape of intra-tumoral heterogeneity. Compared to previous WGS studies in this field, the improved resolution attained here supports the validity of the somatic variants identified in this analysis.

Among the variations exclusively identified in malignant MGTs, seven genes were associated with non-synonymous mutations (*KCNK2*,* AHCTF1*,* YTHDC1*,* GSDME*,* FKBP14*,* HECW1*,* HECTD4*). Several of these genes have been reported in previous research of their roles and functions. In this study, the *HECTD4* gene displayed common missense mutations in all malignant MGTs, and amino acid substitutions were confirmed to be “probably damaging.” E3 ubiquitin ligases such as *HECTD4* play a critical role in recognizing specific proteins and attaching ubiquitin, a process that is also vital in disease states. For instance, abnormalities in this ubiquitination process can affect the abnormal growth or survival of neoplastic cells in diseases such as cancer. Although there has been no research to date on the role of *HECTD4* in breast cancer, relevant studies have suggested that other similar genes in the *HECT* family, such as *HECTD1* and *HECTD3*, play significant roles in breast cancer progression and treatment response [47, 48]. Similar to these genes, mutations in *HECTD4* might trigger abnormalities in ubiquitination in malignant MGTs. *KCNK2* is known to be highly expressed in the central nervous system, and various studies have performed on the role of *KCNK* genes in breast cancer treatment and prevention strategies [49, 50]. Furthermore, *KCNK2* has been evaluated as a prognostic factor in various tumors, and high expression in breast cancer has been associated with improved overall survival rates [51]. In this study, a single base deletion was confirmed in the 3’-UTR coding sequence of *KCNK*2. It is thought that the gene expression might have been affected by deletions, potentially correlating with poor survival rates. As this is only found in malignant MGTs, a coding deletion (c.*96delA) at critical transcriptional sites of *KCNK2* gene should be noted. Finally, the *YTHDC1* gene, which recognizes N6-methyladenosine (m6A) RNA modifications, has been identified as a crucial gene related to the metastasis of triple-negative breast cancer in humans. In a previous study, increased expression of *YTHDC1* has been associated with enhanced metastatic capabilities, and cells with high *YTHDC1* levels have been found in metastatic nodules in the lungs of animal models [52]. In current study, the SNV in the 5’-UTR region of the *YTHDC1* gene, which is identified only in malignant tumors, may be associated with increased expression of the gene.

Among the variations identified exclusively in epithelial-derived malignant tissues, excluding myoepithelial cells, ten genes were found to be associated with significant non-synonymous mutations (*SRSF10*,* MPHOSPH10*,* NIPBL*,* KAT7*,* UNC5CL*,* ENSA*,* MUC5B*,* SLITRK1*,* MYH7B*,* PPP1R3F*). Some of these genes have been reported in various studies to be related to the pathogenesis of and to be potential therapeutic targets for human breast cancer. First, the cohesin loading factor, nipped B-like protein (NIPBL), forms an essential complex with sister chromatid cohesion 4 (SCC4) and facilitates the loading of cohesin onto chromatin during DNA replication. This process is critical for maintaining chromosomal stability by ensuring proper sister chromatid cohesion and segregation during the cell cycle [53, 54]. In the current study, a missense mutation in the *NIPBL* gene was identified, with a risk prediction result of “possibly damaging.” In general, *NIPBL* mutants, cohesion complexes form normally but fail to bind to chromosomes [53], potentially affecting their functions in the cell cycle and leading to tumorigenesis [55]. Furthermore, in vitro studies on human breast cancer cell lines have shown that downregulation of *NIPBL* induces apoptosis and autophagy [56]. Considering these findings, the *NIPBL* gene, while correlated with tumorigenesis, may serve as a candidate gene for gene therapy in epithelial-derived malignant MGTs. Similarly, the lysine acetyltransferase 7 (*KAT7*) gene may also be a candidate for gene therapy. *KAT7*, a member of the MYST KAT family, regulates cell survival, DNA replication, and transcription [57–59]. Recent studies in human breast cancer have shown that *KAT7* upregulates phosphoinositide 3-kinase (PIK3CA), leading to activation of the PI3K/AKT signaling pathway, which is known to contribute to cell survival and proliferation. In human medicine, PI3KCA mutations are common in 20–30% of patients with breast cancer, and drugs targeting the PI3K pathway are being developed. Based on these findings, combining targeted therapy against KAT7 with inhibition of the PI3K pathway may offer a promising approach for improving breast cancer treatment. This may be able to apply equally to MGT treatment in dogs. Finally, in this study, the SNV in the *MUC5B* gene identified was found exclusively in epithelial-derived malignant MGTs. This aligns with the fact that the *MUC5B* gene encodes information for mucin, which is known to predominantly be present on the surface of epithelial cells. Overexpression of mucin, which is observed in many cancers, is crucial for cancer cell adhesion, invasion, immune system evasion, and capturing of biological molecules such as growth factors [60]. A study reported high frequencies of MUC5B protein in breast cancer tissues, whereas mucin was not expressed in normal breast samples [61]. In this case, in silico risk analysis assessed the missense mutation’s significance as “unknown,” but the mutation could have affected protein expression. Other research has suggested that overexpression of *MUC5B* could stimulate aggressive tumor cell behavior, increasing cell proliferation, tumor growth, and dissemination, highlighting the need for vaccine development [62]. In this context, the *MUC5B* gene might be considered as a potential candidate gene for targeted therapy in epithelial-derived mammary tumors in both humans and dogs.

Based on previous studies [12–19], 17 genes closely associated with human breast cancer were selected to check for variations in MGTs derived from WGS. Twenty-four non-synonymous mutations were identified at crucial transcriptional sites involving 11 genes (*ESR1*,* CDH1*,* SHBG*,* TP53*,* PALB2*,* ERBB2*,* CDKN2A*,* STK11*,* PGR*,* BRCA2*, and *AR*). Of these mutations, 15 were not found in the blood sample but were present in all seven MGTs. In particular, the *CDH1* gene showed six mutations, including five in the 3’-UTR and one critical deletion mutation related to amino acid loss (p.His130del). Three missense variants were also identified in the *BRCA2* gene, with one variant (c.6686G >T) showing pathogenicity still regarded as “conflicting” in the search for corresponding human amino acid sequence variant (p.Cys2212Phe) in the BRCA2 protein. In this study, that variant was verified in silico to be “probably damaging” (PolyPhen Score = 0.969). This is consistent with previous findings in both human breast cancer and canine MGTs regarding the risk associated with *BRCA2* mutations [63, 64]. Therefore, we propose that the coding variant (c.6686G >T) may be associated with the pathogenicity of canine MGT. Coding mutations found in *ERBB2* (c.3565G >A) and *CDKN2A* (c.376G >T) were aligned with human sequences in ClinVar, and both were deemed of uncertain significance. PolyPhen verification for these amino acid changes showed “benign” outcomes, suggesting that these two mutations might have lower significance in canine MGTs.

Out of the 24 mutations identified, 9 were found exclusively in several tumors based on histopathological examinations. In particular, a missense variant (c.685T >C) in the *TP53* gene was detected only in malignant myoepithelioma (grade 3). This mutation was verified by PolyPhen as “probably damaging” (PolyPhen Score = 1.000), and searches in the ClinVar database for the aligned human amino acid reported it as “likely pathogenic”. This result is consistent with existing research suggesting that *TP53* gene mutations are more common in higher-grade breast cancers and are associated with more aggressive tumor characteristics and a poor prognosis [65]. Similarly, a missense mutation in the *PGR* gene was observed exclusively in a benign tumor, namely, a lipoma. This mutation was also identified as “probably damaging” (PolyPhen score = 1.000) by in silico verification. To our knowledge, there have been no studies demonstrating a link between the *PGR* gene and the development of lipomas, making it difficult to establish a causal relationship. We are able to predict that the influence of a single *PGR* gene is likely outweighed by a combination of various factors. An amino acid deletion (p.Glu761*) in the CDH1 protein was found only in several types of MGT tissues (lipoma, tubulopapillary carcinoma, tubular carcinoma). According to the ClinVar database, the loss of glutamic acid at that position is verified as “pathogenic” by an expert panel review in human breast cancer. These results also show that there are limitations in existing studies with regard to explaining why mutations were found only in several types of tumors but not all. Therefore, further studies are needed to elucidate the underlying mechanisms based on the findings of this research.

In our analysis, several non-synonymous variants (e.g., in *CDH1*, *BRCA2*, and *SHBG*) were consistently observed across multiple histopathologically distinct tumors from the same dog. Given the shared germline background, these recurrent variants could represent early driver events that arose prior to divergence into distinct histological subtypes. Alternatively, such variants may have emerged independently in separate tumors through convergent evolution under similar selective pressures, or they may reflect field cancerization effects within the mammary gland tissue. While our high sequencing depth and stringent variant filtering reduce the likelihood of technical artifacts, they cannot be completely excluded. Distinguishing between a common clonal origin and independent tumor evolution would require further analyses, such as phylogenetic reconstruction based on variant allele frequencies or spatially resolved sequencing. Future studies incorporating these approaches will be critical to elucidating the temporal and evolutionary context of shared somatic mutations.

Integrating histopathologic classification with genomic data revealed several patterns. The *TP53* missense variant (p.Ser229Pro, probably damaging) was uniquely detected in the malignant myoepithelioma (Grade 3), suggesting a potential role in late-stage tumor progression in myoepithelial neoplasms. In contrast, the *NIPBL* missense mutation (p.Val469Leu) was restricted to epithelial-derived malignant carcinomas, potentially reflecting involvement in chromosomal cohesion defects characteristic of glandular malignancies. *HECTD4* missense variants (p.Ala1955Thr) were consistently present across all malignant tumors, irrespective of histologic subtype, implying a broader role in malignant transformation. Although limited to a single case, these observations support the hypothesis that certain genomic alterations align with histologic subtype or grade, providing a framework for future multi-case studies to validate these relationships.

This study has several limitations. First, as the study involved comparisons of tumors derived from a single case, it was not possible to employ statistical methods. While genetic homogeneity among all MGTs from a single case could be assured, it is impossible to exclude individual specificity of the genetic information. Second, verification of the identified mutations through methods such as Sanger sequencing or functional experiments could not be conducted due to insufficient remaining sample volumes. As a result, our findings are based solely on high-coverage WGS and in silico predictions. Further studies with experimental validation are warranted to support the clinical and biological relevance of these mutations. Third, the actual changes of the protein affected by mutations were not verified but were predicted based on computer software. Further studies using RNA-seq are required to validate the expression levels induced by the mutations identified in this study. Fourth, as normal tissue was unavailable in this case, WGS analysis on normal tissue could not be performed. Instead, a blood sample was analyzed. Finally, a fifth limitation of our study is that our analysis was intentionally focused on the high-confidence detection of SNVs and small insertions/deletions, and did not include a formal analysis of larger structural variations such as copy number variations (CNVs). These include highly variable sensitivity and elevated false-positive rates as mentioned in previous research [66, 67]. Therefore, to maintain a uniform and high standard of evidence for all reported variants, we prioritized the accuracy of our primary findings in the SNV landscape over a more comprehensive but potentially less reliable multi-variant analysis. Future studies employing dedicated CNV-optimized pipelines on a larger cohort of cases will be invaluable for elucidating the full spectrum of structural alterations in these tumors and would be a valuable complement to the foundational work presented here.

Despite these inevitable limitations, our study offers important implications that strongly encourage large-scale follow-up investigations. In addition to demonstrating the consistency of mutations in key human orthologs in canine MGTs, our results provide species-specific insights that missense mutations in *HECTD4* and *NIPBL* may serve as candidate driver events with canine-specific relevance. Therefore, a further larger longitudinal study of canine patients with intra-individual heterogeneity would contribute to more comprehensive understanding of clonal evolution, tumor progression, and subclonal dynamics. Moreover, follow-up studies elucidating clinical relevance (e.g., recurrence risk, metastasis potential) and biological mechanisms associated with these key mutations would improve the clinical and translational potential of naturally occurring canine mammary tumors in comparative oncology.

## Conclusions

This study presents the first high-resolution WGS analysis comparing somatic mutations across multiple histopathological subtypes of MGTs in a single dog. Unlike previous studies that examined single tumors from various breeds to explore germline risk factors or parallels with human breast cancer, our approach reveals subtype-specific mutations and tumor evolution within a shared genetic background. Notably, we confirmed mutations in known cancer genes such as *BRCA2* and *TP53*, and also identified potentially novel, canine-specific drivers—*HECTD4* in malignant tumors and *NIPBL* in epithelial subtypes—suggesting their role in tumor development and progression. Taken together, this unique case study offers novel insights into the genomic heterogeneity, clonal evolution, and subtype-specific pathogenesis of naturally occurring canine MGTs. These findings underscore the need for future large-scale, longitudinal studies to further elucidate the clinical and biological relevance of key somatic alterations, including their associations with metastatic potential, recurrence, and therapeutic responsiveness. Ultimately, our results support the translational value of canine models in comparative oncology and highlight their potential to inform both veterinary and human breast cancer research.

## Supplementary Information

Supplementary Material 1

## Data Availability

The variant data for this study have been deposited in the European Variation Archive (EVA) at EMBL-EBI under accession number PRJEB76921 (https://www.ebi.ac.uk/eva/?eva-study=PRJEB76921).
