# Supplementary material for: Comparative genomic analysis of multiple mammary tumors from a single dog: whole-genome sequencing study
Source: Ir Vet J. 2025 Oct 30;78:27. doi: 10.1186/s13620-025-00311-5 (PMC12573984; doi:10.1186/s13620-025-00311-5)
Supplement: Supplementary file 1 — Supplementary Material 1 [file 13620_2025_311_MOESM1_ESM.docx]

**Supplementary Table 1.** Histopathological findings in seven MGT tissues

| Sample | Histopathological findings | Diagnoses |
| --- | --- | --- |
| T1 | - The tumor lesion was mainly composed of spindle‐shaped cells or round cells admixed with an extracellular fibrillar basophilic myxoid matrix. - The outgrowing myoepithelial were oval to spindle-shaped. It had poorly demarcated cell borders, a moderate amount of eosinophilic or basophilic cytoplasm, with occasional intracytoplasmic clear vacuoles. - Nuclei were round, central, with finely stippled chromatin and a single nucleolus. - Around thick population of myoepithelial cells was a scant, variably basophilic, fibrillar material (myxoid matrix), which was also found within vacuoles in the cytoplasm of the neoplastic cells. - Neoplastic cells exhibited severe anisokaryosis and anisocytosis and mitotic figures were frequently found. | Malignant myoepithelioma (Grade III of malignancy) |
| T2 | - The tumor lesion was composed of well-differentiated lipocytes. | Lipoma |
| T3 | - Tumor cells were arranged predominantly as tubular or gland‐like structures. - The lining of the tubules was usually 1–2 cells thick. - Nuclear pleomorphism was mild with vesicular nuclei with margination of the chromatin or clumped chromatin and with a single or indistinct nucleolus. - Mitotic activity were very low. | Tubulopapillary carcinoma (Grade I of malignancy) |
| T4 | - Tumor cells were arranged predominantly as tubular or gland‐like structures. - Tubules were lined by hyperchromatic cuboidal cell with 1–2 cells thickness. - Nuclei were mostly hyperchromatic with mild nuclear pleomorphism, but, some of which had hypochromatic nuclei with margination of the chromatin and clumped chromatin filling the nucleus. - Cytoplasm was eosinophilic cytoplasm and cell margins are relatively distinct. - There was multiple bone formations. | Tubular carcinoma (Grade I of malignancy) |
| T5 | - The tumor lesion had two different cell populations as followings;   ◼ The first population of epithelial cells was arranged in irregular tubules that were lined by a single to several layers of cuboidal to columnar cells with a scant, moderate amount of eosinophilic cytoplasm.  ◼ The second population was myoepithelial cells propagating within the interstitium, arranged in irregular bundles within a fibrillar basophilic matrix.   - Neoplastic cells had poorly demarcated cell borders, scant to moderate, homogeneous, slightly eosinophilic cytoplasm, round to ovoid central nuclei with finely stippled chromatin and a small central nucleolus with moderate anisokaryosis and anisocytosis. - Mitotic figures were rarely found. | Complex carcinoma (Grade II of malignancy) |
| T6 | - The neoplastic lesion consisted of well demarcated, non‐infiltrative nodule(s) that have not extended through the basement membrane into the surrounding mammary tissue. - Lobular hyperplasia was often found in multiple areas. - Tumor lesion was densely cellular and consisted of closely packed cells arranged in irregular tubules, nests and cords. - Tumor cells varied from polygonal to round to cuboidal, often had a scant amount of eosinophilic cytoplasm and a high nuclear‐to‐cytoplasmic ratio. - Nuclei were often hyperchromatic, central, round to oval, with clumped chromatin and a single nucleolus. | *In situ* carcinoma (Grade I of malignancy) |
| T7 | - Tumor cells were arranged predominantly as tubular structures. - Most tubules were lined by uniform, hyperchromatic cuboidal cell with 1–2 cells thickness within the surrounding basement membrane. - There was a follicular cyst, especially infundibular cyst lined by squamous epithelium which keratinized through the prior formation of a granular layer. | Simple adenoma |

**Supplementary Table 2.** Genomic features of SNVs identified only in MGT tissues

| **Genomic feature** | **SNV counts** |
| --- | --- |
| Intron_variant | 344,363 |
| Intergenic | 315,831 |
| Upstream_gene_variant | 93,588 |
| Downstream_gene_variant | 44,876 |
| 3_prime_UTR_variant | 6,678 |
| Synonymous_variant | 2,892 |
| Intragenic_variant | 2,709 |
| Missense_variant | 2,241 |
| 5_prime_UTR_variant | 1,630 |
| Non_coding_transcript_exon_variant | 940 |
| Splice_region_variant&intron_variant | 680 |
| 5_prime_UTR_premature_start_codon_gain_variant | 257 |
| Non_coding_transcript_variant | 198 |
| Splice_region_variant&non_coding_transcript_exon_variant | 132 |
| frameshift_variant | 127 |
| Splice_region_variant&synonymous_variant | 67 |
| Splice_region_variant | 57 |
| Missense_variant&splice_region_variant | 53 |
| Splice_donor_variant&intron_variant | 40 |
| Disruptive_inframe_deletion | 35 |
| Splice_acceptor_variant&intron_variant | 33 |
| Frameshift_variant&splice_region_variant | 30 |
| Stop_gained | 28 |
| Splice_acceptor_variant & splice_region_variant  & 3_prime_UTR_variant & intron_variant | 16 |
| Conservative_inframe_deletion | 15 |
| Disruptive_inframe_insertion | 14 |
| Stop_lost | 9 |
| Start_lost | 7 |
| Gene_fusion | 7 |
| Conservative_inframe_insertion | 7 |
| Splice_donor_variant&splice_region_variant&intron_variant | 5 |
| Splice_acceptor_variant & splice_region_variant  & intron_variant & non_coding_transcript_exon_variant | 4 |
| Splice_acceptor_variant&splice_region_variant&intron_variant | 3 |
| Splice_donor_variant&splice_region_variant&intron_variant  & non_coding_transcript_exon_variant | 3 |
| Stop_retained_variant | 3 |
| Frameshift_variant&start_lost | 2 |
| Frameshift_variant&stop_gained | 2 |
| Stop_lost&splice_region_variant | 1 |
| Start_retained_variant | 1 |
| Start_lost&splice_region_variant | 1 |
| Splice_donor_variant & splice_region_variant  & 5_prime_UTR_variant & intron_variant | 1 |
| Initiator_codon_variant | 1 |
| Frameshift_variant & splice_donor_variant  & splice_region_variant & intron_variant | 1 |
| Conservative_inframe_insertion & splice_region_variant | 1 |
| **Total** | **817,589** |

**Supplementary Table 3.** Similar variants in dogs corresponding to human amino acids with the major variants reported in the ClinVar database.

| **#Chrom** | | **Pos** | **Gene** | **AA_change**  **(Canine)** | **AA**  **(Corresponding**  **human)** | **AA_change**  **(Identified in ClinVar)** | **Mutation ID** | **Classification** | **Review status*** |
| --- | --- | --- | --- | --- | --- | --- | --- | --- | --- |
| 5 | 81347396 | | *CDH1* | p.Glu761* | p.Glu758 | p.Glu758Lys | 186218 | Likely pathogenic | ★ |
|  |  |  |  |  |  | p.Glu758Gly | 1205648 | Uncertain significance | ★★ |
| 5 | 81368793 | | *CDH1* | p.His130del | p.His128 | p.His128Tyr | 439043 | Uncertain significance | ★★ |
|  |  |  |  |  |  | p.His128Pro | 2442989 | Uncertain significance | ★ |
|  |  |  |  |  |  | p.His128Arg | 956509 | Uncertain significance | ★ |
|  |  |  |  |  |  | p.His128Gln | 2453340 | Uncertain significance | ★ |
| 5 | 32746330 | | *SHBG* | p.Arg153Cys | p.Arg82 | NA | NA | NA | NA |
| 5 | 32771285 | | *TP53* | p.Ser229Pro | p.Ser241 | p.Ser241fs | 2679237 | Likely pathogenic | ★ |
|  |  |  |  |  |  | p.Ser241* | 2584754 | Likely pathogenic | ☆ |
|  |  |  |  |  |  | p.Ser241Tyr | 376663 | Pathogenic | ★★ |
|  |  |  |  |  |  | p.Ser241Cys | 177791 | Pathogenic | ★★ |
|  |  |  |  |  |  | p.Ser241Phe | 12359 | Pathogenic | ★★ |
|  |  |  |  |  |  | p.Ser241fs | 1414190 | Pathogenic | ★ |
|  |  |  |  |  |  | p.Ser241Thr | 959232 | Pathogenic | ★ |
|  |  |  |  |  |  | p.Ser241fs | 958618 | Pathogenic | ★ |
|  |  |  |  |  |  | p.Ser241delinsThr | 644381 | Pathogenic | ★ |
|  |  |  |  |  |  | p.Ser241Ala | 376665 | Conflicting classifications of pathogenicity | ★ |
| 6 | 22461625 | | *PALB2* | p.Leu480Arg | p.Leu482 | p.Leu482Phe | 923896 | Uncertain significance | ★★ |
| 9 | 22713702 | | *ERBB2* | p.Val1189Ile | p.Val1184 | NA | NA | NA | NA |
| 11 | 41339546 | | *CDKN2A* | p.Ala126Ser | p.Ala134 | p.Ala134Gly | 1737089 | Uncertain significance | ★ |
|  |  |  |  |  |  | p.Ala134Val | 650117 | Uncertain significance | ★★ |
|  |  |  |  |  |  | p.Ala134Pro | 495536 | Uncertain significance | ★★ |
| 20 | 58046465 | | *STK11* | p.Pro275Gln | p.Pro275 | p.Pro275fs | 1762622 | Pathogenic | ★ |
|  |  |  |  |  |  | p.Pro275Ser | 1485942 | Uncertain significance | ★ |
|  |  |  |  |  |  | p.Pro275Leu | 480721 | Uncertain significance | ★★ |
| 21 | 571913 | | *PGR* | p.Leu630Ile | p.Leu624 | NA | NA | NA | NA |
| 25 | 7822932 | | *BRCA2* | p.Cys2229Phe | p.Cys2212 | p.Cys2212fs | 52141 | Pathogenic | ★★★ |
|  |  |  |  |  |  | p.Cys2212fs | 2096859 | Pathogenic | ★ |
| 25 | 7825345 | | *BRCA2* | p.Thr1425Pro | p.Thr1430 | p.Thr1430Ala | 495463 | Conflicting classifications of pathogenicity | ★ |
|  |  |  |  |  |  | p.Thr1430Ser | 967743 | Conflicting classifications of pathogenicity | ★ |
|  |  |  |  |  |  | p.Thr1430Ile | 231592 | Conflicting classifications of pathogenicity | ★ |
| 25 | 7839968 | | *BRCA2* | p.Ile103Thr | p.Val109 | p.Val109Phe | 1025967 | Uncertain significance | ★ |
|  |  |  |  |  |  | p.Val109Ile | 921408 | Uncertain significance | ★★ |
|  |  |  |  |  |  | p.Val109Ala | 1729616 | Uncertain significance | ★ |
| X | 52313056 | | *AR* | p.Leu605Ile | p.Leu617 | p.Leu617Pro | 458360 | Likely pathogenic | ★ |

Review status* (★★★★: Practice guidelines; ★★★: Reviewed by an expert panel; ★★: Criteria provided, multiple submitters, no conflicts; ★: Criteria provided, single submitter; ☆: No assertion criteria provided
